# Genomic analysis of WD40 protein family in the mango reveals a TTG1 protein enhances root growth and abiotic tolerance in *Arabidopsis*

**DOI:** 10.1038/s41598-021-81969-z

**Published:** 2021-01-26

**Authors:** Lin Tan, Haron Salih, Nwe Ni Win Htet, Farrukh Azeem, Rulin Zhan

**Affiliations:** 1 Hainan Key Laboratory of Banana Genetic Improvement, Haikou Experimental Station, Chinese Academy of Tropical Agricultural Sciences (CATAS), Haikou, 571101 Hainan China; 2 Crop Sciences, Faculty of Agriculture, Zalingei University, Central Darfur, Sudan; 3 Microbiology Laboratory, Biotechnology Research Department, Kyaukse, 05151 Myanmar

**Keywords:** Plant evolution, Plant genetics

## Abstract

WD40 domain-containing proteins constitute one of the most abundant protein families in all higher plants and play vital roles in the regulation of plant growth and developmental processes. To date, WD40 protein members have been identified in several plant species, but no report is available on the WD40 protein family in mango (*Mangifera indica* L.). In this study, a total of 315 WD40 protein members were identified in mango and further divided into 11 subgroups according to the phylogenetic tree. Here, we reported mango TRANSPARENT TESTA GLABRA 1 (*MiTTG1*) protein as a novel factor that functions in the regulation of Arabidopsis root growth and development. Bimolecular fluorescence complementation (BiFC) assay in tobacco leaves revealed that *MiTTG1* protein physically interacts with *MiMYB0*, *MiTT8* and *MibHLH1*, implying the formation of a new ternary regulatory complex (MYB-bHLH-WD40) in mango. Furthermore, the *MiTTG1* transgenic lines were more adapted to abiotic stresses (mannitol, salt and drought stress) in terms of promoted root hairs and root lengths. Together, our findings indicated that *MiTTG1* functions as a novel factor to modulate protein–protein interactions and enhance the plants abilities to adjust different abiotic stress responses.

## Introduction

WD40 repeat domains (WD40 domain-containing proteins) play important roles in diverse biological functions such as cytokinesis, cell division, apoptosis, meristem organization, floral development, cytoskeleton assembly, protein trafficking, chromatin modification, and gene transcriptional mechanism^1–3^. WD40 proteins are characterized as core residues of certain conserved motifs (44–60 amino acid) with glycine-histidine (GH) at the N-terminus and tryptophan-aspartic acid (WD) pair at the C terminus^4^. The name of WD40 proteins originate from the conserved WD regions and the core residue length of a single repeat contains about 40–60 amino-acids^5,6^. WD40 proteins are highly prevalent in all eukaryotes and rarely found in prokaryotic organisms^7^. WD40 protein motifs fold into seven-bladed β-propeller domain repeats which act as a scaffold for various protein–protein interactions and form functional complexes^1^. Generally, the functions of WD40 proteins need to be assisted in protein–protein interactions, which have no intrinsic enzymatic activities^8^. The interaction between WD40 repeat proteins, MYB and bHLH transcription factors have been widely investigated in flavonoid biosynthesis^9^. Also, the TTG1-bHLH-MYB complex is directly involved in the regulation of root growth and trichome patterning on the *Arabidopsis* leaf^10^. Several WD40 repeat proteins are associated with epidermal traits in *Arabidopsis*, Perilla and maize^11^. Recently, in *Arabidopsis* GIGANTUS1 is connected with biomass accumulation and seed germination through a protein–protein interaction^7^. WD40-REPEAT 5a plays an important role in drought tolerance through the regulation of nitric oxide accumulation^12^. Another WD40 protein (XIW1) is directly involved in the association of the stability ABI5 and ABA responses in *Arabidopsis*^13^. *TaWD40D* is significantly associated with wheat responses to abiotic stress^14^. Highlighting the features of WD40, its significance and relevance in the study of mangoes have to be elaborated. Previously, the genome-wide identification of the WD40 protein family has been carried out in *Arabidopsis*^4^, cotton^15^, foxtail millet^2^, rice^16^, wheat^17^, human^18^ and peach^19^. Whereas, no study has been performed to identify these proteins in mango (*Mangifera indica* L.), which is one of the most important tropical fruit with high economic value worldwide. Mango belongs to the Anacardiaceae family, also known as the “king of fruits”, and is a fruit with great dietary value^20–22^. The nutrient supply of mango includes calories, vitamins, minerals and fiber^23^. Agro-industrial residues and the flesh of mango have some bioactive compounds, containing nutrient and non-nutrient elements with biological properties that act mostly through redox mechanisms^20^. Each mango fruit breed varies in shape, color, size, taste, flavor and fiber content^24^. The exocarp part grows into a leathery protective skin which is smooth, waxy and green, containing lenticels that emerged from stomata^22^. Based on cultivars, once mango is ripe, the fruit skin changes to a yellow sparsely marked with red or pale green^23^. In this study, we identified 315 WD40 protein members in mango at a genomic analysis level, including their domain numbers, evolutionary relationship, gene location and predicted molecular functions. Besides, we reported the molecular cloning of cDNA encoding a mango WD40 protein, *MiTTG1* (*Mi01g20920*), an ortholog of the *Arabidopsis AT5G24520* (TTG1) which promotes root growth and enhances osmotic, salt and drought stress tolerance in *Arabidopsis*. This work will provide a solid platform to better understand the functional roles of *MiTTG1* gene and will help further investigation on the biological function and molecular mechanisms of these proteins family in mango.

## Results and discussion

### Identification, sequence characterization, subcellular localization prediction of the mango fruit WD40 repeat proteins

In order to identify the members of the WD40 protein family in the mongo genome database^25^, blast profiles (HMM research and local blast) were used as a query within the mango genome project. We identified 485 protein sequences in the mango genome database. After removing redundant sequences based on the Perl program, WD40 candidate protein sequences were investigated manually with the SMART online and Pfam database for the presence of the WD40 domain (Supplementary Table 1). Finally, a total of 315 protein members were identified in the mango genome database, which varied from 1 to 12 repeat domains (Supplementary Table 1). However, only a single copy of WD40 protein, *MiTTG*1 (four repeat domains) was identified to be the ortholog of *Arabidopsis* TTG1 in the mango genome database. In recent years, several WD40 proteins that are orthologs to the *Arabidopsis* TTG1 gene have been identified in some plant species, such as cotton (*Gossypium hirsutum*), *Prunus persica*, *Punica granatum* and maize (*Zea mays*)^26–29^. In studied plant species, It was found that there was only one copy of gene-encoding WD40 repeat protein (TTG1)^26–29^. Besides the divergence of conserved WD40 repeat domains, the protein encoded by mango WD40 gene family were significantly varied in the numbers of amino acids, physicochemical properties and divergence in the subcellular localization within a cell (Supplementary Table 1). The amino acid sequences of the 315 mango WD40 protein sequences varied in lengths from 84 to 3604 amino acids, with an average length of 655.4 amino acids (Supplementary Table 1). To explain the potential functions of the proteins encoded by the WD40 gene family under investigation, understanding their physiochemical features are vital, for example, the proteins can be divided depending on their molecular mass (size) and isoelectric point (charge) properties and their abundance then determined subsequently^30^. The molecular weights were largely varied between 9644.89 and 400,731.34 Da (Dalton) with an average of 72,516.59 Da. The investigation of the subcellular localization of 315 mango WD40 protein members was predicted by WoLF PSORT online tool. The result revealed that 166 of WD40 proteins were localized in the nucleus, 78 in chloroplasts and 43 in the cytosol while the remaining 28 WD40 proteins were localized in various subcellular membranes, such as mitochondria, cytoskeleton, peroxisome and plastid (Supplementary Table 1). This finding implies that WD40 proteins might be involved in regulating various physiological features of plant growth and developmental processes under different environmental conditions. The silico mapping of *MiWD40*s on chromosomes showed an uneven distribution of the genes on all the 20 chromosomes of mango. Three hundred and four (304) WD40 genes were distributed across 20 chromosomes in the mango genome while 11 genes were mapped to the scaffold (unknown chromosome). Some mango chromosomes and chromosomal positions had a high density of WD40 gene members while others do not (Supplementary Fig. 1). The highest densities of WD40 genes were detected on chromosome 3 with 34 genes, and the lowest densities of WD40 genes were showed on chromosomes 13 and 15 with 7 genes for each.

### Phylogenetic tree and conserved motif analysis, GO annotation of the mango WD40 proteins family

**Figure 1:**
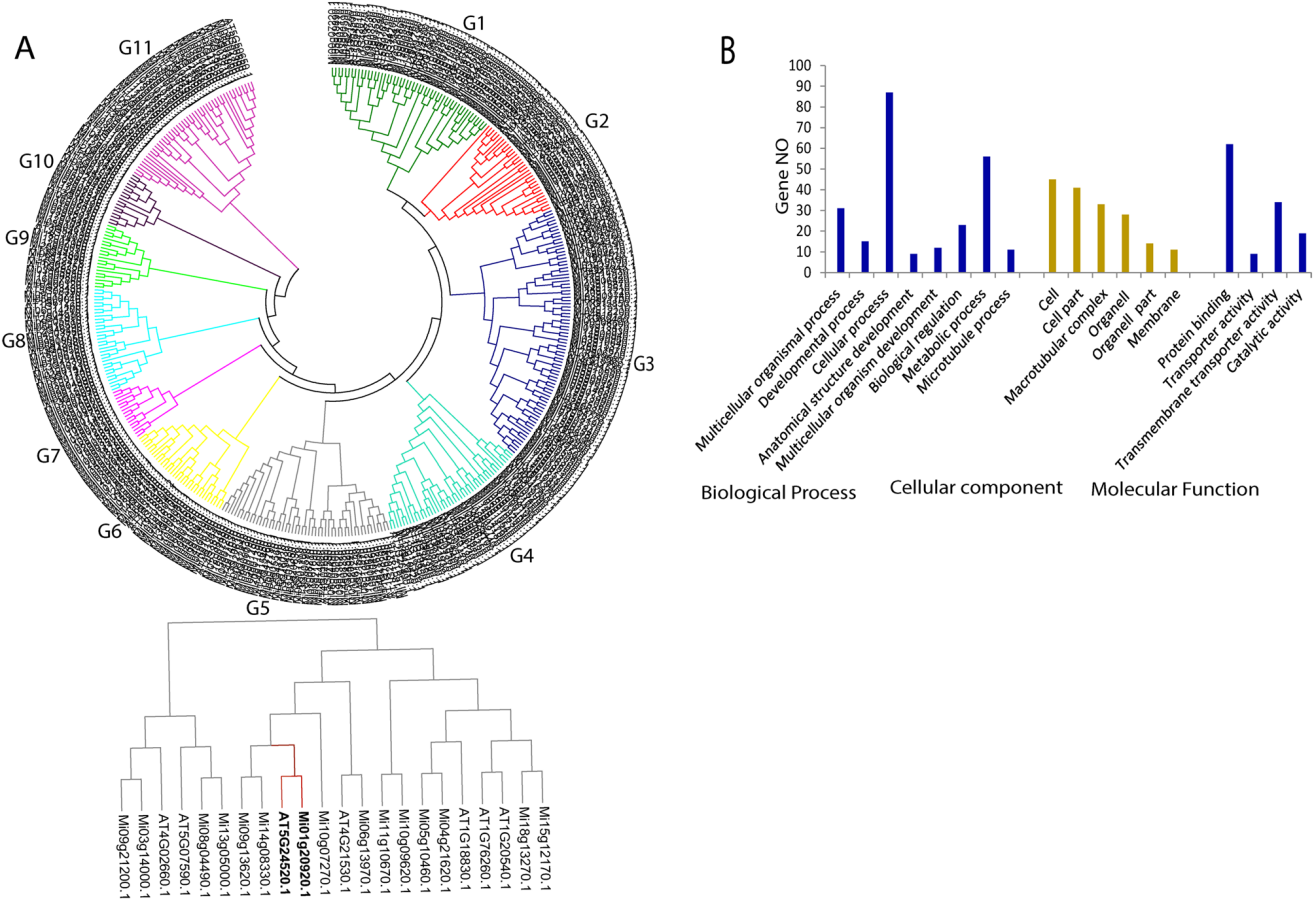
Functional analysis of mango WD40 proteins family. (**A**) Phylogenetic tree relationship between mango and *Arabidopsis* of WD40 proteins was conducted by MEGA 6.0 using the Neighbor-Joining (NJ) method. The11 subgroups are shown in colors. (**B**) GO annotation analyses of mango WD40 proteins were examined for their functions in biological process, molecular functions and cellular components.

The evolutionary relationship analysis of the mango WD40 protein family was performed by the construction of an un-rooted phylogenetic tree using the NJ (neighbor-joining) method, with 1000 bootstrap replicates. According to the protein sequence similarities, the 315 mango and 107 *Arabidopsis* WD40 protein members were divided into 11 subgroups, which varied in number from 16 to 75 protein members (Fig. 1A). The bootstrap value for several subgroups of the neighbor-joining tree was not high as a result of reasonably great numbers of WD40 protein sequences that were also mentioned in earlier reports^2,15,31,32^. Most mango WD40 proteins showed high similarities within subgroups of phylogenetic tree analysis. This result was in agreement with previous reports in cotton and foxtail millet^2,15^. WD40 proteins family from mango and *Arabidopsis* are consistently present at intervals around the phylogenetic tree analysis. Specifically, based on the protein sequence similarities, each mangoWD40 repeat protein has a matching homolog in *Arabidopsis thaliana*. This result is consistent with the previous findings which showed that the majority of *Arabidopsis* WD40 proteins have a corresponding homolog WD40 protein in the plant kingdom^4^. Further investigation was done to predict the multiplicity of conserved motifs within 315 *MiWD40* proteins using the MEME program. This analysis identified ten conserved motifs that were labeled as motif 1 to motif 10 (Supplementary Fig. 2). The result revealed that motif 1 is the most conserved motifs among the ten motifs, being present in all mangoWD40 proteins. Motifs 2 and 4 were in the second and third most abundant motifs, respectively (Supplementary Fig. 2). Most WD40 proteins within the same subgroups had a common motif in terms of motif distribution and composition, which indicated the WD40 protein members within a given subgroup, may have similar functional roles. Furthermore, a certain unique conserved motif was detected in a particular subgroup, which revealed a stronger sign of involvement of this motif in specific roles within the higher plants. The gene ontology (GO) annotation was conducted using the OmicsBox/Blast2Go (https://www.blast2go.com/) tool, and it predicted the potential function of WD40 proteins. Mango WD40 proteins were categorized into three groups by GO analysis based on their involvement in biological process, cellular component and molecular function. Accordingly, the WD40 proteins were categorized into 8 groups of biological processes; a significant number of WD40 proteins were associated with the cellular process, metabolic process, multicellular organismal process, biological regulation and developmental process (Fig. 1B). In terms of the cellular component prediction of WD40 proteins, they were relatively enriched in the cell, cell part, macrotubular complex, organelle, organelle part and membrane. In molecular functions, the WD40 proteins were found to be associated with protein binding, transmembrane transporter activity, catalytic activity and transporter activity (Fig. 1B) which possibly support their involvement in protein–protein interaction networks. In the previous finding, it was noticed that some members of WD40 proteins physically interact with bHLH and MYB transcription factors, which are involved in different aspects of plant growth and development^33–37^.

### *MiTTG1* encoded WD40-repeat protein

**Figure 2:**
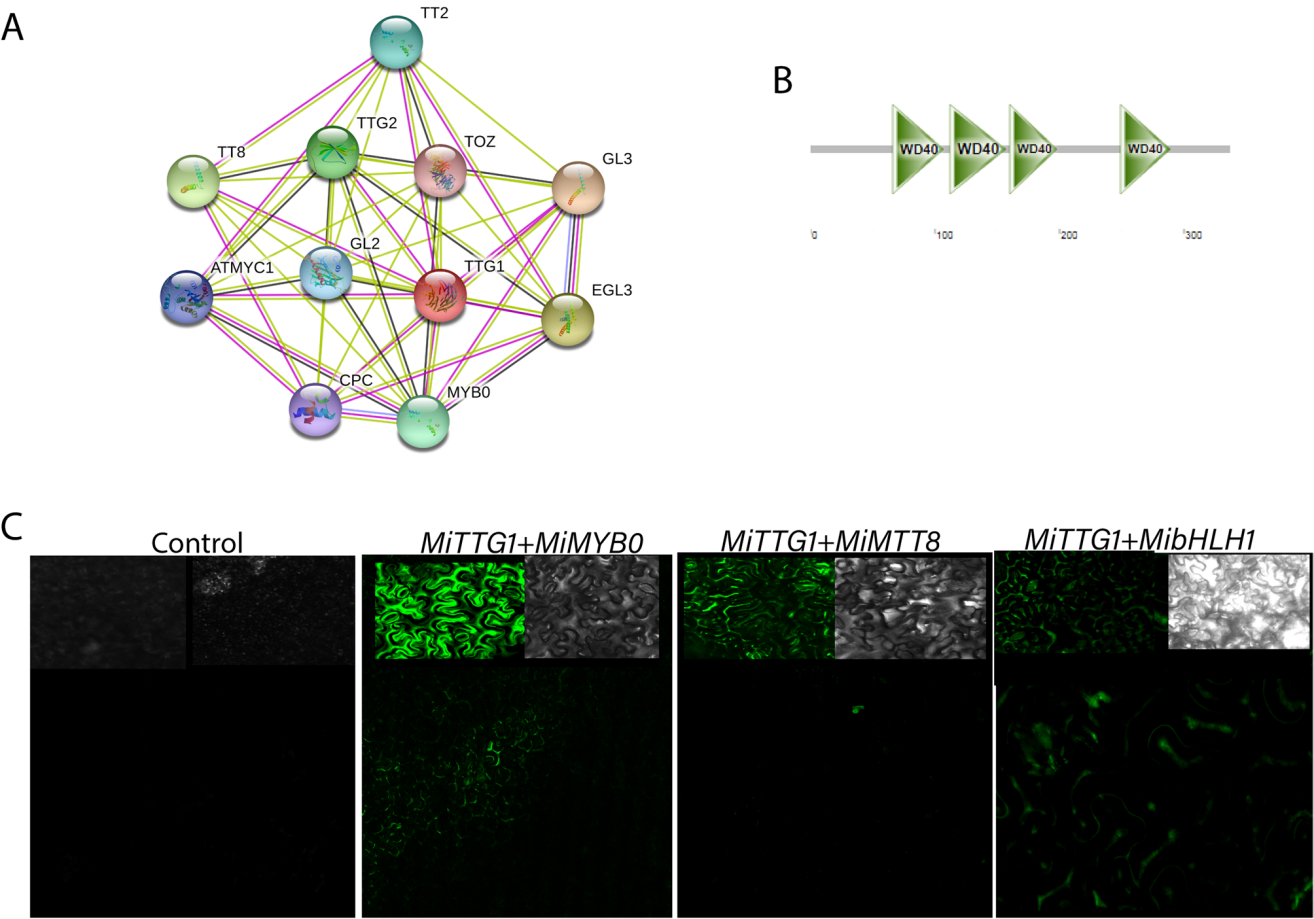
Protein–protein interaction networks of MangoWD40 proteins. (**A**) The prediction of protein interaction networks of WD40 with other transcription factors by STRING database. Each node is represented a protein and each edge is represented an interaction between two proteins. (**B**) Four conserved motifs of *MiTTG1* protein. (**C**) *MiTTG1* physically interacts with *MiMYB, MiTT8* and *MibHLH* transcription factors in tobacco leaves were observed by confocal microscopy.

The mangoTTG1gene (*MiTTG1*), *Mi01g20920* (ortholog of *AT5G24520*) possessed a complete ORF of 1014 bp encoding a protein sequence containing 337 amino acids with a theoretical pI and molecular mass of 8.66 and 67,306.6 Da, respectively (Supplementary Table 1). There were four WD-repeat domains in *MiTTG1* at 66 ~ 107, 112 ~ 157, 160 ~ 198, and 249 ~ 289 amino acids (Fig. 2A). The knowledge about the protein interaction network of the target protein is a key factor in spurring the investigation of the protein involvement in plant growth and developmental processes such as signal transduction, cell formation, pattern establishment, organ development, and plant defense^8,38,39^. To understand protein interaction between TTG1 gene and other proteins in plants, the STRING database (https://string-db.org/) was used to perform the computational analysis of protein–protein interactions (Fig. 2B). The result of functional protein association networks of the mango TTG1 gene showed that this protein can physically interact with the 4 bHLH proteins (TT8, GL3, EGL3 and *ATMYC1*), 3 MYB proteins (MYB0, TT2 and CPC) and 3 other proteins, TTG2 (WRKY), GL2 (Homeobox-leucine zipper) and TOZ (WD40). To get insights into the multi-interaction of *MiTTG1* with other proteins, the BiFC assay was employed to detect physical interaction between *MiTTG1* and 3 proteins (*MiMYB0*, *MiTT8* and *MibHLH1*). The BiFC results showed that GFP was only detected when *MiTTG1* was co-expressed with *MiMYB0*, *MiTT8* and *MibHLH1*, respectively while no GFP was captured when *MiTTG1* was replaced by a free vector (Fig. 2C). The BiFC assay demonstrated the interaction between *MiTTG1* with *MiMYB0*, *MiTT8* and *MibHLH1* in tobacco leaf cells, implying that a new ternary regulatory mango MYB-bHLH-WD40 might be functioned and shaped in a transgenic plant. The study result is consistent with earlier findings of the interaction of WD40-MYB-bHLH proteins in plants^10,11,27,40^.

### *MiTTG1* is involved in the regulation of root growth and development

**Figure 3:**
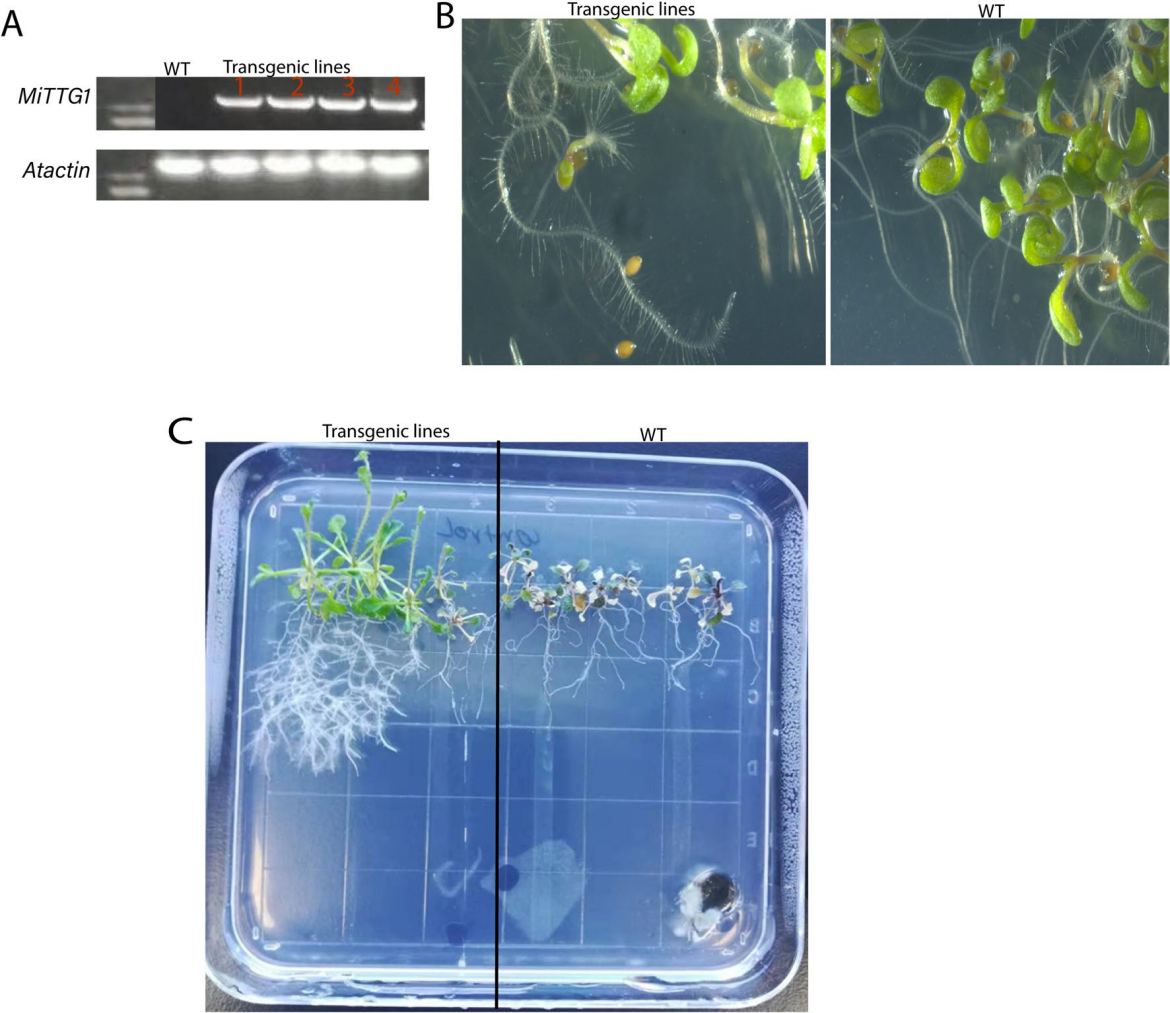
Expression pattern of *MiTTG1* in *Arabidopsis*. The transcripts expression levels of the inserted *MiTTG1* of T1 transgenic *Arabidopsis* lines confirmed by RT-qPCR with biological replicates. (**A**) Phenotypes of root and root hairs of wild-type and transgenic lines after 7 days post germination on ½ MS. (**B**) Phenotypes of root and root hairs of wild-type and transgenic lines after 21 and 30 days post germination on ½ MS.

Root hair systems that root epidermal cells are tubular-shaped and are important for nutrient acquirement, environmental interactions and soil anchorage in higher plants^41^. To elucidate the function of the *MiTTG1* in root development, we systematically investigated the root phenotypes in young *Arabidopsis* transgenic lines (*MiTTG1* was introduced into the wild-type) and wild-type. The transgenic *Arabidopsis* lines containing *MiTTG1* were generated and confirmed by RT-PCR (Fig. 3A). The transgenic lines had higher root hair density than the wild-type plants (Fig. 3B). For 21 day-old plants, the relative root hair density and root lengths were higher and longer in transgenic lines as compared to the wild type (Fig. 3C). Taken together with the above data, this indicates that *MiTTG1* enhances root hair density and root length, which suggests that the increased root system in the *MiTTG1* transgenic line is promoted by its involvement in root growth and development. In *Arabidopsis thaliana, AtTTG1* plays opposite roles in the regulation of root hairs and leaf trichome differentiation^33,37^. The heterologous expression of *LbTTG1* in *Arabidopsis* plants decreased root hair density and increased trichome numbers^42^. The interaction between genetic factors and environmental signals is a key player for the determination of both the length and the abundance of root hairs^43^.

### *MiTTG1* overexpression confers tolerance to mannitol and salt stress during the early stages of root development in *Arabidopsis*

**Figure 4:**
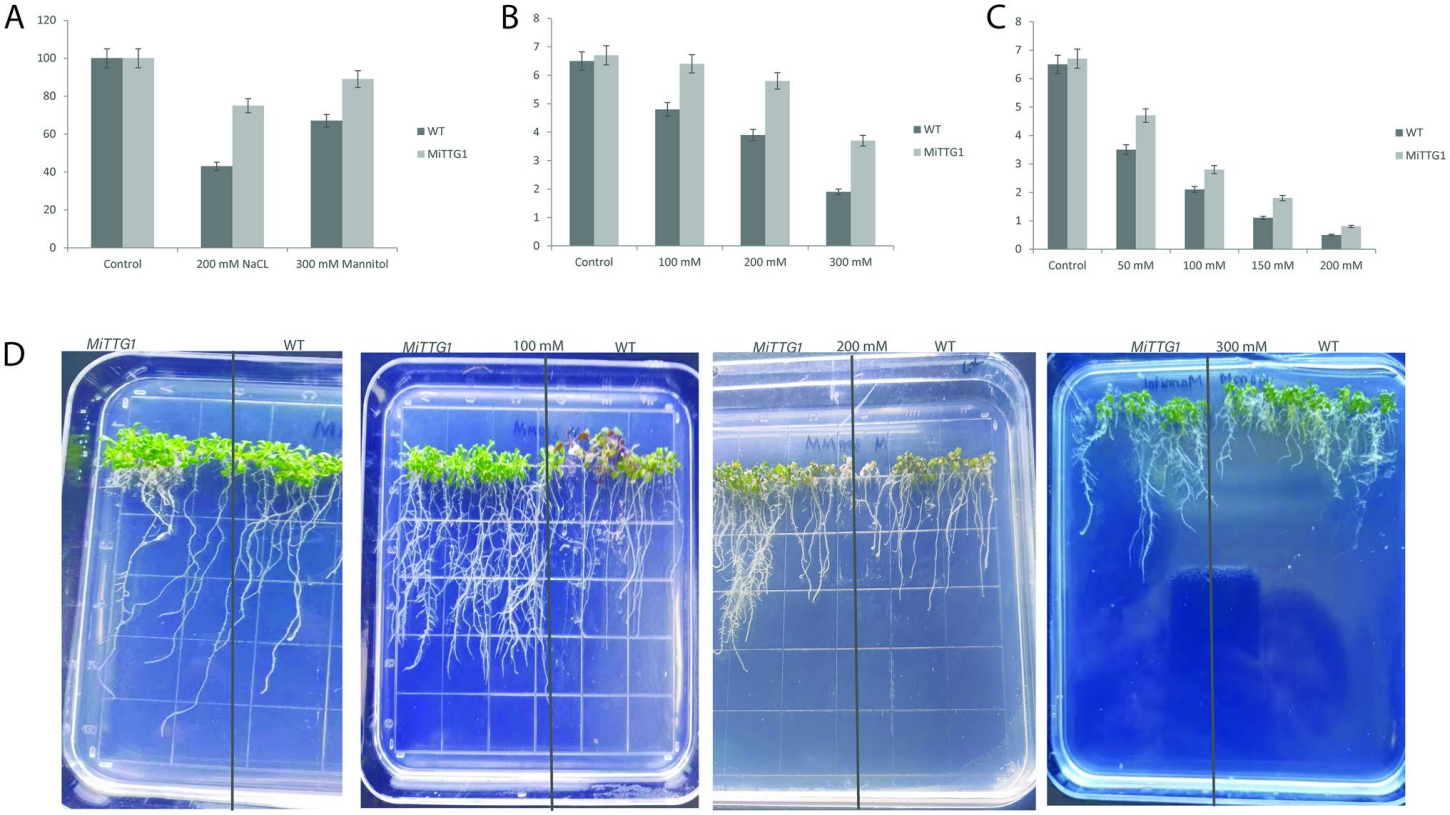
Overexpression of *MiTTG1* enhances abiotic stress tolerance. (**A**) Seed germination rate of transgenic lines and wild type on ½ MS supplemented with 300 mM of mannitol and 200 mM for 5 days. (**B**) Root length comparisons of transgenic lines and wild type on ½ MS supplemented with different concentrations of mannitol (control, 100, 200, and 300 mM) for 21 days. (**C**) Root length comparisons of transgenic lines and wild type on ½ MS supplemented with various concentrations of salt (control, 50, 100, 150, and 200 mM) for 21 days. (**D**) Root elongation and root density comparisons of transgenic lines and wild type on ½ MS complemented with mannitol (control, 100, 200, and 300 mM) for 21 days.

To further examine whether *MiTTG1* is involved in abiotic stress responses in plants, we conducted a reverse genetic method to generate transgenic *Arabidopsis* lines from wild-type (overexpressing *MiTTG1*). The T3 homozygote lines of transgenic *Arabidopsis* had a single copy of *MiTTG1* (Fig. 3A) and were selected for seed germination rate and root phenotypic analysis. The result showed that there was no significant difference in seed germination stage between the transgenic lines and the wild-type under normal conditions on ½ MS (Fig. 4A). In the presence of mannitol and salt, the germination rate of both *MiTTG1*overexpression in transgenic lines and wild type seed was relatively reduced, but the reduction of wild type was greater than that of transgenic seedlings. Under the treatment of 300 mMmannitol, about 89% of the transgenic seeds germinated, while only 67% of wild type seeds germinated at 5 days. Remarkably, 75.3% of the transgenic seeds germinated on ½ MS supplemented with 200 mMNaCl, while only 43.3% of the wild-type seeds germinated after 5 days of treatment (Fig. 4A and Supplementary Table 3). *MiTTG1* overexpression enhanced the tolerance of salt and mannitol within *Arabidopsis* transgenic lines, indicating that WD40 proteins may play a role in coordinating the plant molecular responses to environmental stresses. To analyze the effect of the mannitol and salt treatments on root growth, *MiTTG1* overexpression lines and the wild-type were used against different concentrations of mannitol and salt. The result showed that the root lengths differed between transgenic lines and wild-type plants under the mannitol and salt treatments at 21 days (Fig. 4B,C and Supplementary Table 3). The transgenic lines showed higher root hair density and longer root lengths than in wild type under various concentrations of mannitol treatments (Fig. 4D and Supplementary Fig. 3), demonstrating increased tolerance to osmotic stress. The root lengths significantly increased in transgenic lines by 24%, 20% and 22% with various concentrations of mannitol, 100, 200 and 300 mM, respectively. Under salt treatment 50, 100, 150, 200 mM, the level root lengths increased by 15%, 10%, 10% and 4%, respectively in transgenic lines compared with the wild-type as shown in Supplementary Table 3. The finding of these results demonstrates that *MiTTG1* could play a vital role in plant responses to salt and osmotic tolerance during germination and post-germination stages. Overexpression of *LbTTG1* increased the adaptability of transgenic *Arabidopsis* lines to NaCl treatment^42^^.^

### *MiTTG1* overexpression confers drought and stress-related marker genes

**Figure 5:**
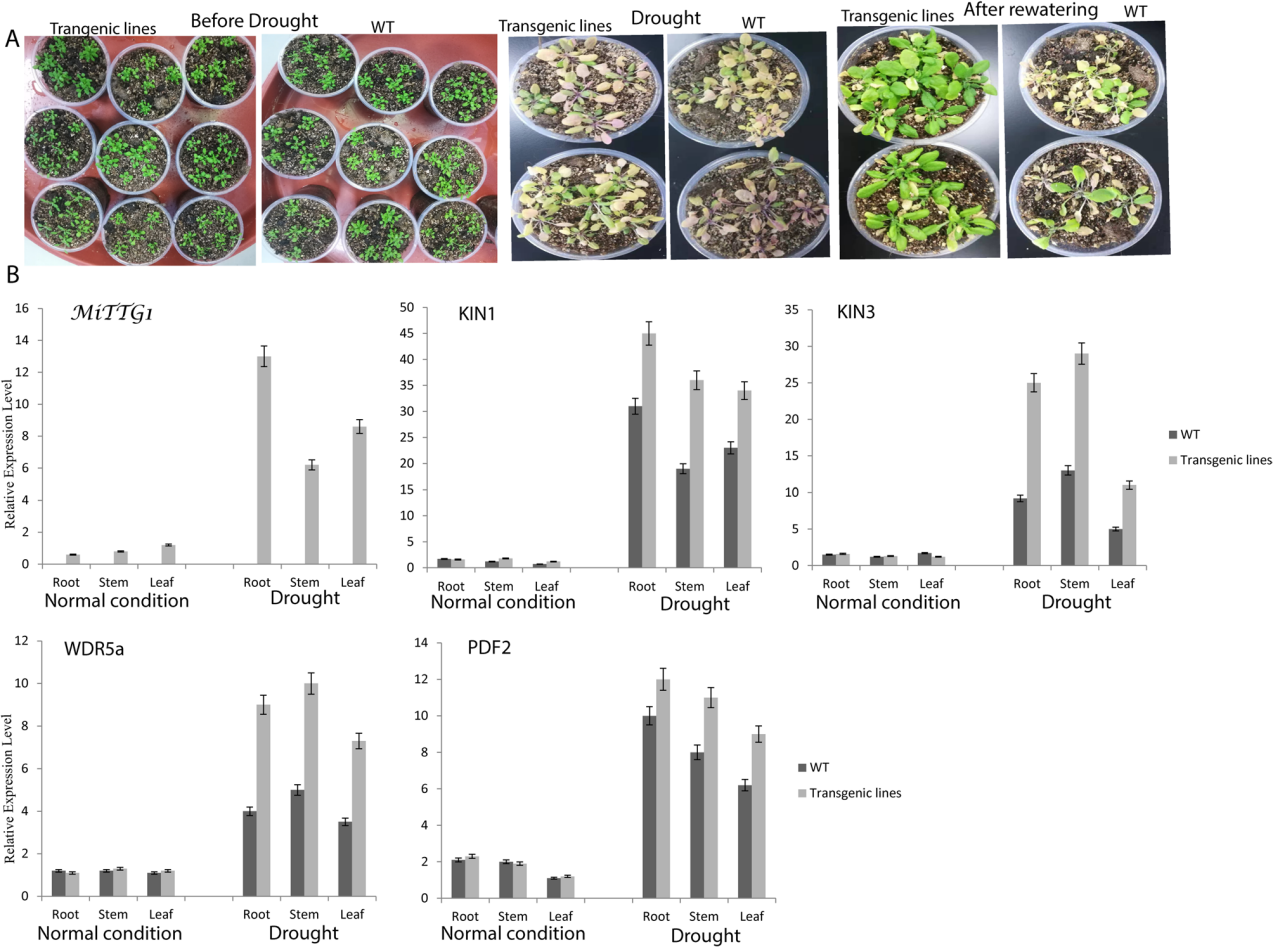
Overexpression of *MiTTG1* enhances drought stress resistance. (**A**) Drought stress treatments of transgenic lines and wild-type. 3 week old plants in soil were subjected to drought stress by withholding water for 14 days and the *Arabidopsis* plants were photographed after rewatering for 4 days. (**B**) Expression levels of *MiTTG1* and stress-related genes (WDR5a, KIN1, KIN3 and PDF2) in transgenic lines and wild type. At actin was used as the gene reference, each experiment was done three times.

In order to understand the possible biological function of the *MiTTG1* gene in the drought stress tolerance, transgenic lines and wild-type plants were exposed to drought situations by withholding water for 14 days (Fig. 5). It was observed that all the transgenic lines recovered while some wild type plants died after rewatering plants for 4 days (Fig. 5A). In general, wild type plants showed a more sensitive phenotype with chlorosis and more wilting of rosette leaves than the transgenic lines (Fig. 5A). It shows that *MiTTG1* might be essential for mechanism-regulated plant stomatal closure in *Arabidopsis* plants in response to drought stress. To investigate the mechanism by which *MiTTG1*overexpression affects the drought tolerance phenotype of transgenic lines and wild type plants, we compared the expression of known stress-related genes, such as WDR5a, KIN1, KIN3 and PDF2 (Fig. 5B). These stress-related genes have been extensively examined in relation to drought stress tolerance in *Arabidopsis*^12,44^. Drought-stress experiment showed that the expression levels of all stress-related genes and *MiTTG1* were highly prompted in the transgenic lines by drought stress as compared to wild type (Fig. 5B), suggesting that overexpression of *MiTTG1* gene has a positive role in enhanced drought stress resistance in these plants. Our findings showed that for the first-time, *MiTTG1* plays a functional role in regulating abiotic stress tolerance in *Arabidopsis*. In other plants, *TTG1* gene controls various features of plant growth and development such as proanthocyanidin and anthocyanin, plant defense^45^, root growth, seed coat pigment accumulation and leaf trichome differentiation^33,37^.

## Conclusion

The WD40 protein family has various copies of the WD40 domain which fold into β-propeller arrangement thus acts as a scaffold for many protein–protein interactions. A total of 315 WD40 proteins were identified in mango genome and divided into 11 subgroups based on phylogenetic analysis. The biological and molecular functional grouping indicated that the mangoWD40 proteins are associating with many cellular functions in higher plants. WD40 protein sequences are mainly localized in the nucleus and cytoplasm while the remaining distributed in other subcellular membranes. The BiFC assay showed that *MiTTG1* physically interacts with *MiMYB0*, *MiTT8* and *MibHLH1* in the tobacco leaf, suggesting that a new ternary regulatory mango MYB-bHLH-WD40 might be formed in transgenic plants. The findings indicate that *MiTTG1* confers *Arabidopsis* improved tolerance to abiotic stress by regulating root growth and development. This first-time analysis of WD40 proteins in mango tropical fruit will provide important evidence of the functional variations among the mango tropical fruit.

## Methods

### Identification of mango WD40 proteins

Mango (*Mangifera indica*) genome assembly and genome annotation sequences were obtained from NCBI-BioProject (https://www.ncbi.nlm.nih.gov/bioproject/PRJNA487154)^25^ for further comprehensive analysis. The HMM (Hidden Markov Model) folder of the WD40 protein domain (PF00400.31) was obtained from the Pfam (http://pfam.xfam.org/) database^46^ and was used as a query to search against the mango genome using HMMER software (version 3.1b2; http://hmmer.org/) with a default E-value < 10^−10^. The consistent protein sequences from *Arabidopsis*, peach and cotton species were obtained from the TAIR database (TAIR; http://www.Arabidopsis.org/), Phytozome database (https://phytozome.jgi.doe.gov/) and cottongen website (https://www.cottongen.org), respectively and also were used as a query to search against mango genome database using local blast tool with a default E-value < 10^−10^. The Perl program was used to remove the redundant sequences among the identified gene sequences. The SMART database (http://smart.embl-heidelberg.de/) and the Pfam database (http://pfam.sanger.ac.uk/) were also used to ensure all mango WD40 protein sequences contained the WD40 domain. Only the mango protein sequences with WD40 domain were used for further investigations. Furthermore, the ExPASy Server tool (http://web.expasy.org/compute_pi/) was applied to predict the molecular weights and of isoelectric points WD40 proteins. The WoLFPSORT database (http://wolfpsort.org/) was also used to examine the subcellular localization of *MiWD40* proteins. The chromosomal positions of WD40 members were done through blastN queries against the mango genome. MapChart software was used to generate a physical map of *MiWD40* genes.

### Phylogenetic analysis and conserved motif distribution and Gene Ontology (GO) Annotation of WD40 proteins in mango

The phylogenetic analysis of WD40 protein sequences was performed using multiple sequence alignments of mango and *Arabidopsis* WD40 protein members with ClustalW (http://www.ebi.ac.uk/Tools/msa/clustalw2/). MEGA 6.0 software (http://www.megasoftware.net) was used to generate an unrooted phylogenetic tree analysis using N-J ((Neighbor-Joining)) method with a bootstrap of 1000 replicates, p-distance and pairwise deletion. To predict the features of homologous WD40 domain and the occurrence of the common amino acids detected at each location in every repeat of the *MiWD40* protein domains, we searched common motifs shared by WD40 protein sequences by uploading their sequences to the online tool Multiple Expectation Maximization for Motif Elucidation (MEME) system (Version 4.9.1, http://meme.nbcr.net/meme/)^47^. The parameters were set as follows: any number of repetitions, the optimum width from 6 to 250 and the maximum number of conserved motifs-10. The functional analysis of *MiWD40* protein sequences was conducted by OmicsBox/Blast2GO (https://www.blast2go.com/) based on their biological process, cellular components and molecular functions. The amino acid sequence of every *MiWD40* protein was incorporated by way of a fast format in the OmicsBox/Blast2Go online tool and blastp against protein sequence of NCBI, annotation and mapping to examine a protein function was done. The WD40 proteins were analyzed by Interpro in OmicsBox/Blast2Go and GO terms were combined with the annotated sequence mentioned previously. To gain more insight into the regulatory role of *MiWD40* proteins in biological function in protein–protein interaction, the STRING online database (https://string-db.org/)^48^ was used to conduct the protein interaction networks of TTG1 protein.

### Plant materials

The seeds of *Arabidopsis thaliana* (wild-type, Col-0) used in this work were obtained from *Arabidopsis* Biological Research Centre (ABRC)*.* These seeds were planted at 22 °C in a growth chamber with a 16-h/8-h light/dark cycle. 5-day-old seedlings grown on half-strength Murashige and Skoog medium (½ MS) plates were transplanted into the ½ MS and soil in a growth chamber at 22 °C in a 16-h/8-h light/dark cycle. Tobacco plants were also used in this study for protein–protein interaction networks. Tobacco seeds were grown into the soil in a growth chamber at 22 °C under a 16-h-/8-h light/dark cycle. Five-week-old plants were infiltrated using *Agrobacterium tumefaciens* strain GV3101.

### Vector construction and transformation of *Arabidopsis thaliana*

The mango TTG1 ORF was inserted into NC frame of pCAMBIA1304-vector by nimble cloning system described by Dr Yan et al.^49^ under the control of the CaMV 35S promoter (p35S:: *MiTTG1*) using primers pNC-*TTG1*-UF and pNC-*TTG1*-UR (Supplementary Table 2A) according to the nimble cloning system (Supplementary Table 2B). The p35S::*MiTTG1* vector was introduced into *Agrobacterium tumefaciens* strain GV3101 and plated on LB agar supplemented with rifampicin (50 µg/mL) and kanamycin (50 µg/mL). A single kanamycin-resistant colony was isolated and used to start a 250-mL culture in LB liquid media supplemented with rifampicin (50 µg/mL) and kanamycin (50 µg/mL), which was incubated for 2 days at 28 °C with checking at 200 rpm until OD (optical density) of 1.0–1.5 at 600 nm (OD600). Bacterial cells were pelleted by centrifugation at 5000 rpm for 30 min and the supernatant discarded. The bacterial pellet was resuspended in 200 mL of 5% sucrose with 0.05% Silwet L77). *Arabidopsis* wild type (Col-0) was used for transformation, which was conducted using the floral dip method described by Clough and Bent^50^. T0 seeds were harvested upon the maturation stage and transgenic lines (T1) were identified by selection on 1/2 MS plates supplemented with Hygromycin (25 µg/mL) and RT-PCR. The homozygous T3 generation was used for further genetic analyses.

### Bimolecular fluorescence complementation (BiFC) assay

Wild type *Nicotiana benthamiana* was used to investigate transient expression following an agro-infiltration procedure that was done according to a published method^49^. The full-length CDS of mango *MiTTG1*, *MiMYB*0, *MiTT8* and *MibHLH1* genes were cloned into the pNC-BiFC vector that contained of two NC frames with various adapters. Four genes of interest flanked by the adapters were recombined into the vector by the Nimble Cloning System. The genes encoding *MiTTG1*, *MiMYB*0, *MiTT8* and *MibHLH1* were amplified with nimble cloning primers containing the adapters in NC frames (Supplementary Table 2A). The PCR products were introduced into the pNC-BiFC vector by Nimble Cloning System. The empty vectors and the recombinant plasmids were transformed into *A. tumefaciens* GV3101 using the freeze–thaw method and were transiently expressed in tobacco leaves by agro-infiltration. Two days after infiltration, the green fluorescent protein (GFP) signals were examined with a FluoView FV1000 confocal microscope (Olympus, Japan) in the transfected cells.

### Abiotic stress analysis of transgenic plants

*Arabidopsis* wild-type (col-0) and homozygous T3 generation transgenic lines were used in abiotic stress experiments. For germination rate, 300 mM/L of mannitol and 200 mM/L salt were used to examine the effect abiotic stress on transgenic lines and wild type seeds. For each replicate 100 seeds were used to investigate the effect. For stress treatment, 3-day-old sterilized seedlings were transferred into ½ MS supplemented with mannitol (0, 100, 200 and 300 mM/L) and NaCL (0, 50, 100, 150 and 200 mM/L). Three weeks later, root phenotypic traits were analyzed. Drought treatment was also done to 3-week-old seedlings in soil with sufficient water by withholding watering for 14 days. Samples from transgenic lines and wild-type were collected from root, stem and leaves. Three biological replicates were conducted to guarantee results reliability. Experimental datasets were presented in the form of the mean of three values with the standard deviation ± SD. The analysis for significance was performed using Student’s t-test.

### Plant materials, RNA isolation and qRT-PCR analysis

To detect the *MiTTG1* overexpression, *Arabidopsis* samples were collected from transgenic lines and wild-type (col-0) from root, stem and leaves. RNA was extracted from Arabidopsis root, stem and leaves using the Tiangen (RNA Aprep Pure Plant Kit). The concentration and quality of RNA samples were tested using a NanoDrop 2000 spectrophotometer and gel electrophoresis. RNA samples with high quality were treated with DNase I (TaKaRa, Japan) to remove genomic DNA contamination. The cDNA was synthesized using the ReverTra Ace qPCR RT kit (TOYOBO, Japan) depending on the manufacturer’s manual. qRT-PCR experiments were performed to measure the expression pattern of TTG1 and related marker genes (WDR5a, PIN1, PIN3 and PDF2) for drought. The qRT-PCR investigation was done using the Applied Biosystems 7500 Real-Time PCR-system and the SYBER premix ExTaq kit (TaKaRa. Japan). The amplification of the target gene was estimated by the SYBR Green fluorescence signal. The Arabidopsis constitutive β-actin was used as a reference gene and specific TTG1 and related marker genes primers were used for qRT-PCR. The primers used in this study were listed in Supplementary Table 2C. The following thermal cycle settings of qRT-PCR and the expression analysis of genes were performed according to Salih et al.^15^.

## Supplementary Information

Supplementary Figure 1.

Supplementary Figure 2.

Supplementary Figure 3.

Supplementary Table 1.

Supplementary Table 2.

Supplementary Table 3.

Supplementary Legends.

## Data Availability

All related data are available within the manuscript and its additional files.
