# Supplementary figures and images for: Genomic analysis of WD40 protein family in the mango reveals a TTG1 protein enhances root growth and abiotic tolerance in Arabidopsis
Source: Sci Rep. 2021 Jan 26;11:2266. doi: 10.1038/s41598-021-81969-z (PMC7838414; doi:10.1038/s41598-021-81969-z)

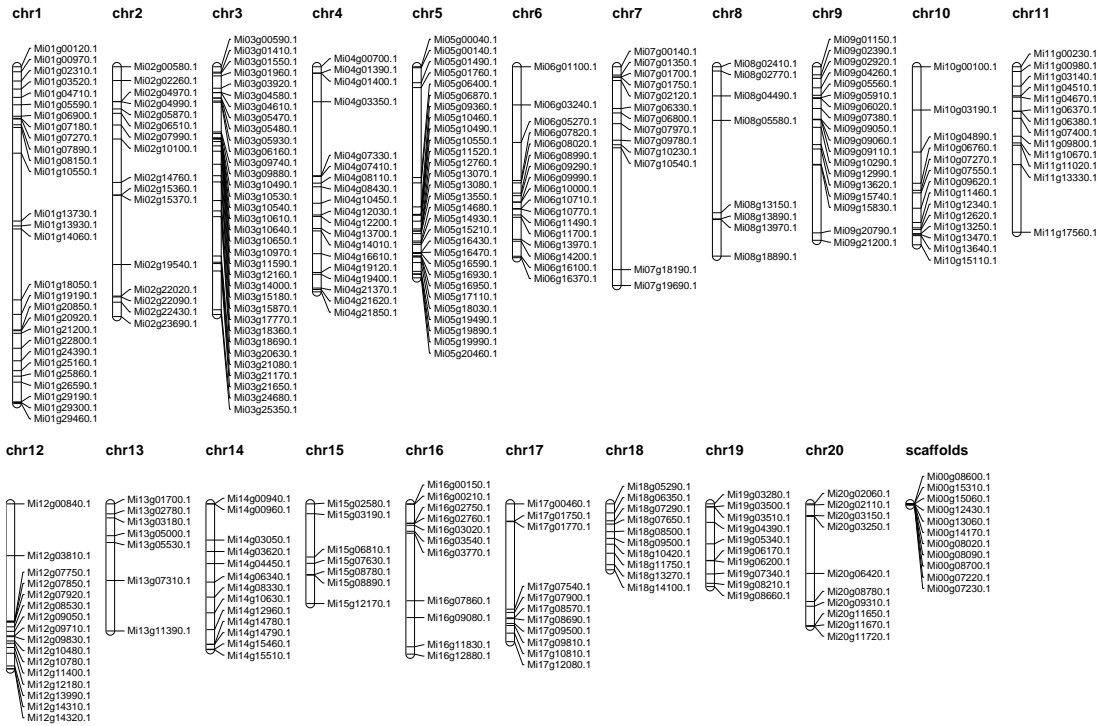

Supplement: Supplementary file 1 — Supplementary Figure 1. [file 41598_2021_81969_MOESM1_ESM.pdf]

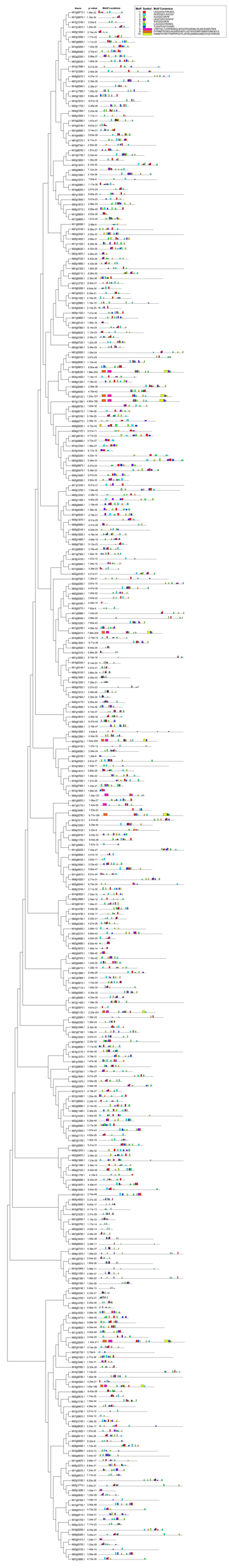

Supplement: Supplementary file 2 — Supplementary Figure 2. [file 41598_2021_81969_MOESM2_ESM.pdf]

Supplementary figure 3: Root growth of transgenic lines and wild type

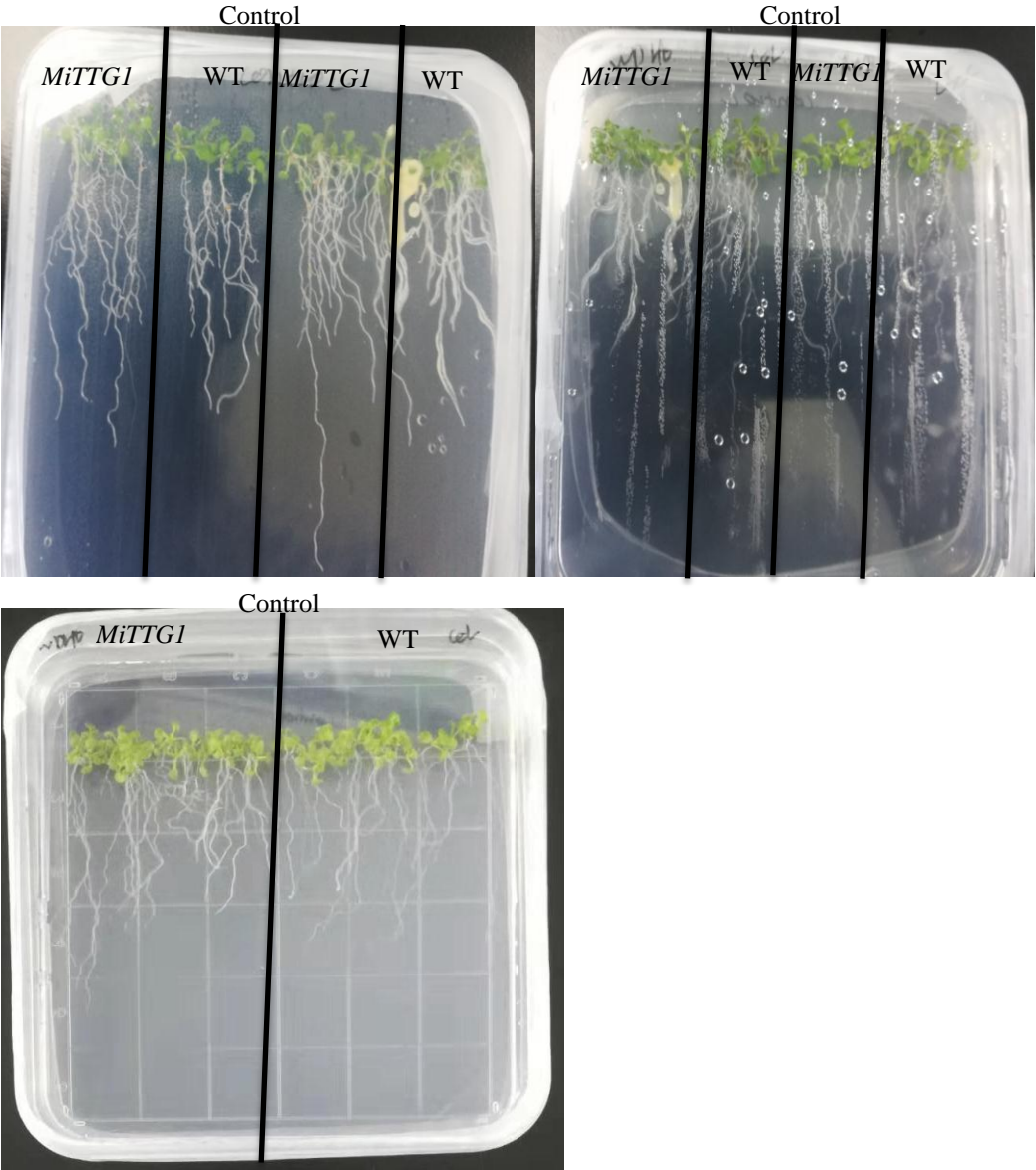

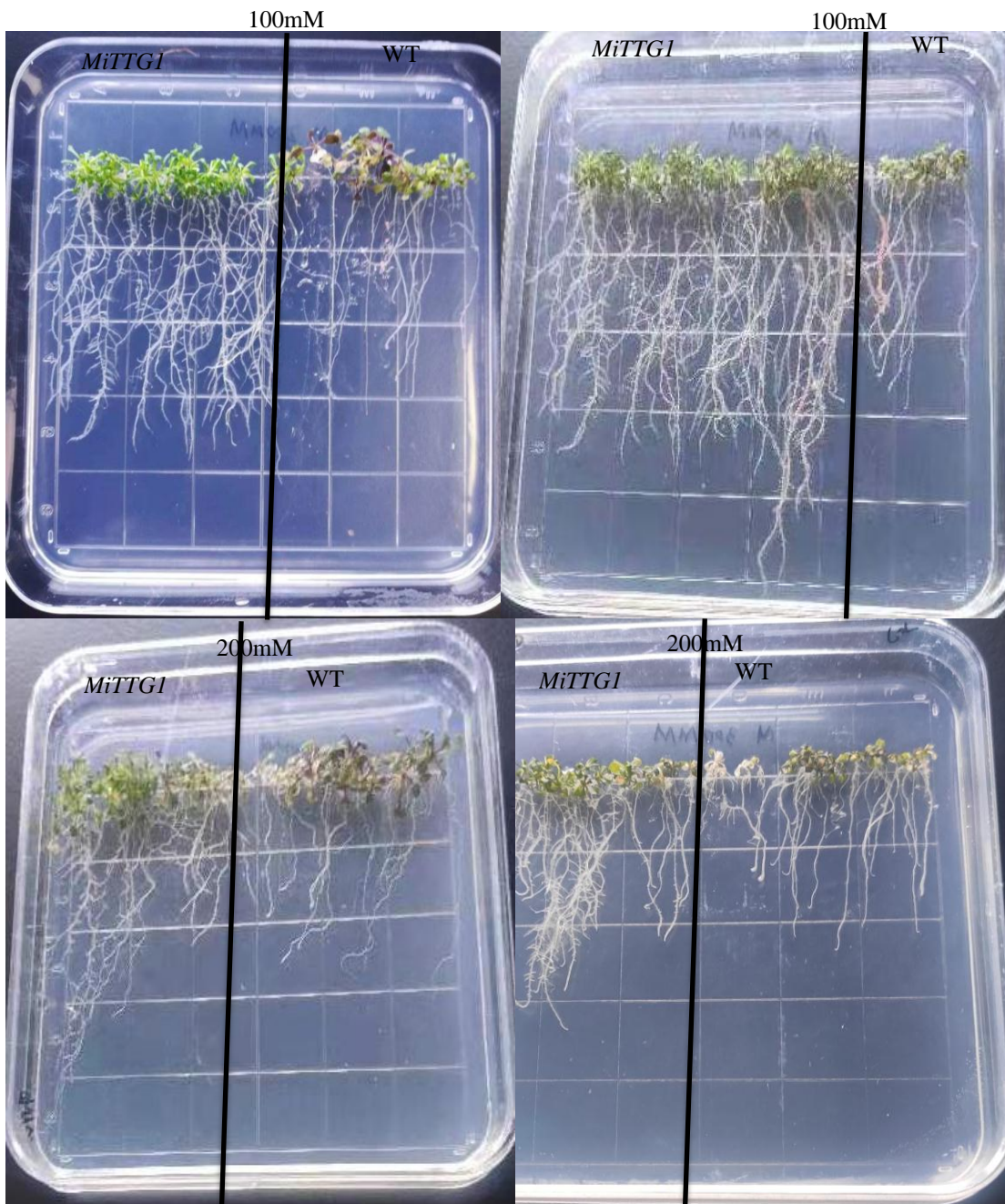

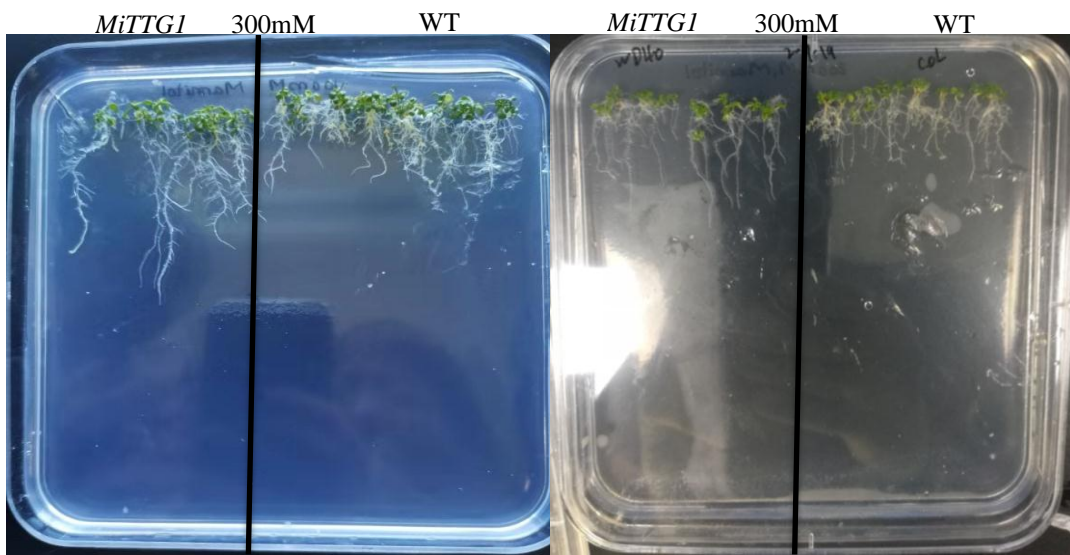

Supplement: Supplementary file 3 — Supplementary Figure 3. [file 41598_2021_81969_MOESM3_ESM.pdf]
