# Supplementary material for: Genomic analysis of WD40 protein family in the mango reveals a TTG1 protein enhances root growth and abiotic tolerance in Arabidopsis
Source: Sci Rep. 2021 Jan 26;11:2266. doi: 10.1038/s41598-021-81969-z (PMC7838414; doi:10.1038/s41598-021-81969-z)
Supplement: Supplementary file 7 — Supplementary Legends. [file 41598_2021_81969_MOESM7_ESM.docx]

**Genomic analysis of WD40 protein family in the mango reveals a TTG1 protein enhances root growth and abiotic tolerance in *Arabidopsis***

Lin Tan^1^, Haron Salih^1,2*^, Nwe Ni Win Htet^1,3^, Farrukh Azeem^1^ and Rulin Zhan^1*^

1. Haikou Experimental Station, Chinese Academy of Tropical Agricultural Sciences (CATAS)-Hainan Key Laboratory of Banana Genetic Improvement, Haikou 571101, Hainan, China.

2. Crop Sciences, Faculty of Agriculture, Zalingei University, Central Darfur, Sudan.

3. Microbiology Laboratory, Biotechnology Research Department, Kyaukse 05151, Myanmar.

* Correspondence should be addressed to Haron salih [salih234@yahoo.com](mailto:salih234@yahoo.com) & Rulin Zhan [zhanrulin555@163.com](mailto:zhanrulin555@163.com)

**1. Lin Tan**

Email: tanlin@catas.cn

Address: Haikou Experimental Station, Chinese Academy of Tropical Agricultural Sciences (CATAS)-Hainan Key Laboratory of Banana Genetic Improvement, Haikou 571101, Hainan, China.

2**. HARON Salih**

Email: [salih234@yahoo.com](mailto:salih234@yahoo.com)

Address: Haikou Experimental Station, Chinese Academy of Tropical Agricultural Sciences (CATAS)-Hainan Key Laboratory of Banana Genetic Improvement, Haikou 571101, Hainan, China.

Crop Sciences, Faculty of Agriculture, Zalingei University, Central Darfur, Sudan.

**3. Nwe NiWin Htet**

Email: nweniwinhtet@gmail.com

Address: Haikou Experimental Station, Chinese Academy of Tropical Agricultural Sciences (CATAS)-Hainan Key Laboratory of Banana Genetic Improvement, Haikou 571101, Hainan, China.

Microbiology Laboratory, Biotechnology Research Department, Kyaukse 05151, Myanmar

4. **Farrukh Azeem**

Email: azeuaf@hotmail.com

Address: Haikou Experimental Station, Chinese Academy of Tropical Agricultural Sciences (CATAS)-Hainan Key Laboratory of Banana Genetic Improvement, Haikou 571101, Hainan, China.

5. **Rulin Zhan**

Email: [zhanrulin555@163.com](mailto:zhanrulin555@163.com)

Address: Haikou Experimental Station, Chinese Academy of Tropical Agricultural Sciences (CATAS)-Hainan Key Laboratory of Banana Genetic Improvement, Haikou 571101, Hainan, China.

**Supplementary files**

**Supplementary Table 1**: Identification of mangoWD40 gene family, amino acids length, protein domain, sub-molecular localization, isoelectric point (pI), molecular weight and chromosomal positions.

**Supplementary Table 2**: Primer sequences were used in this work. (A) Nimble cloning primers with specific gene primer for construction vectors. (B) Some information about Nimble Cloning protocol. (C) RT-qPCR Primers for TTG1 and genes related to drought.

**Supplementary table 3:** Statistical analysis of germination rate and root lengths.

**Supplementary Figure 1**: The chromosomal positions of WD40 genes were mapped to the mango genome.

**Supplementary Figure 2**: Phylogenetic tree relationship, conserved motifs of mangoWD40 proteins family (A) the phylogenetic tree of WD40 proteins in mango (B) Ten conserved motifs of mangoWD40 proteins were identified by MEME are represented by colored boxes and consensus sequences.

**Supplementary figure 3:** Root growth of transgenic lines and wild type.
